# Heme oxygenase-1 polymorphisms associate with ischemic cardiac complications and all-cause mortality in type 1 diabetes

**DOI:** 10.1186/s12933-025-02895-2

**Published:** 2025-08-18

**Authors:** Heli Segersvärd, Niina Sandholm, Valma Harjutsalo, Heidi Tikkanen, Riikka Kosonen, Mika Laine, Ilkka Tikkanen, Per-Henrik Groop, Päivi Lakkisto, the FinnDiane Study Group

**Affiliations:** 1 Minerva Foundation Institute for Medical Research, Biomedicum Helsinki 2, Helsinki, Finland; 2 Research Program for Clinical and Molecular Metabolism, Faculty of Medicine, University of Helsinki, Helsinki, Finland; 3 Folkhälsan Research Center, Haartmaninkatu 8, 00290 Helsinki, Finland; 4 Department of Nephrology, University of Helsinki and Helsinki University Hospital, Helsinki, Finland; 5 Department of Cardiology, Heart and Lung Center, University of Helsinki and Helsinki University Hospital, Helsinki, Finland; 6 Department of Diabetes, Central Clinical School, Monash University, Melbourne, VIC Australia; 7 Baker Heart and Diabetes Institute, Melbourne, VIC Australia; 8 Department of Clinical Chemistry and Hematology, University of Helsinki and Helsinki University Hospital, Helsinki, Finland

## Abstract

**Background:**

Heme oxygenase 1 (HO-1), encoded by the *HMOX1* gene is a highly inducible enzyme with multiple cardiovascular protective properties. Polymorphisms of the *HMOX1* gene, especially a guanine-thymine dinucleotide repeat polymorphism (GTn), affects its transcriptional activity and is associated with cardiovascular complications in the general population. We studied the association of *HMOX1* polymorphisms and HO-1 serum concentrations with vascular complications and all-cause mortality in individuals with type 1 diabetes (T1D).

**Methods:**

The study population consists of individuals with T1D participating in the Finnish Diabetic Nephropathy Study (FinnDiane). We genotyped the *HMOX1* GTn repeat (*n* = 3990), extracted from genome-wide genotyping data two single nucleotide polymorphisms (SNPs) (-413A/T upstream variant rs2071746, and + 99G/C p.Asp7Asn missense variant rs2071747; *n* = 4278), and measured the serum HO-1 concentrations (*n* = 861) from blood samples taken during their study visit. The GTn repeats were divided into short (S) and long (L) alleles where the cutoff point was L ≥ 30 repeats.

**Results:**

In men, the LL genotype was associated with ischemic cardiac events (LL 22.9% vs. SS/SL 17.0%, *p* = 0.001) and all-cause mortality (*p* = 0.031). The association was detected in all individuals (LL 19.5% vs. SS/SL 16%, *p* = 0.006) but not in women (LL 15.7% vs. SS/SL 14.9%, *p* = 0.657). For the -413A/T SNP, men with the AA genotype experienced ischemic cardiac events more frequently (21.0% vs. 17.4%, *p* = 0.044), but no differences were found for women or for men and women together. There were no differences between different genotypes of the + 99G/C variant regarding cardiovascular complications. Also, there was no difference in HO-1 serum concentrations between different genotypes (GTn repeat, -413A/T or + 99G/C). Men had higher HO-1 serum concentrations compared to women (3.12 ± 1.23 ng/ml vs. 2.64 ± 1.04 ng/ml, *p* < 0.001). In women, higher HO-1 serum concentrations were associated with cardiovascular disease and need for antihypertensive and lipid lowering medications.

**Conclusions:**

The LL genotype of the *HMOX1* GTn repeat and the AA genotype of -413A/T SNP were associated with ischemic cardiac complications and all-cause mortality in men, but not in women. Thus, the *HMOX1* genotype may influence the development of cardiovascular complications in individuals with T1D in a sex-dependent manner.

**Supplementary Information:**

The online version contains supplementary material available at 10.1186/s12933-025-02895-2.

## Introduction

Heme, the product of erythrocyte destruction, is extremely toxic and proinflammatory [1]. Therefore, mechanisms to degrade excess heme are essential. Heme oxygenase (HO) catalyzes the degradation of heme to biliverdin, which is then reduced to bilirubin, carbon monoxide, and free iron [2]. There are two isoforms of HO, namely HO-1 and HO-2, encoded by the *HMOX1* and *HMOX2* genes. The highly inducible HO-1 and its byproducts are cytoprotective and therefore potential targets for therapeutic applications [3, 4].

Several studies suggest that polymorphisms in the promoter area of the *HMOX1* gene affect its transcriptional activity and are associated with a wide range of pathological conditions. The most studied *HMOX1* polymorphism in cardiovascular disease (CVD) is the guanine-thymine dinucleotide repeat polymorphism (GTn). Also, a single nucleotide polymorphism (SNP) in the *HMOX1* promoter, —413A/T, has been associated with CVD, but the data are scarce and conflicting [5–9]. Another described SNP is the + 99G/C transversion in the exon area of the *HMOX1* gene, but its clinical relevance is unclear [10, 11].

The GTn repeat lengths in the *HMOX1* promoter area are divided into short (S) and long (L) alleles, or in some studies, into short (S), medium (M), and long (L) alleles, but there is no consensus on the cutoff points. Furthermore, the allele distributions differ between populations, and the populations studied are also quite diverse [8, 12]. In humans, some studies have shown that the long GTn repeat (L-allele, defined as greater than 25–27 repeats) is associated with lower HO-1 inducibility [13–19].

GTn polymorphism has been linked to CVD in many studies. Two systematic reviews reached similar conclusions in 2016. Daenen et al. showed in their meta-analysis (41 studies with a total population of 27,450 participants) that the SS allele was more common in the Asian than in the Caucasian population. Individuals carrying the LL genotype had higher odds of CVD than those carrying the SL or SS genotypes, especially in the Asian populations [12]. Zhang and coworkers concluded in their meta-analysis including 23 studies that the SS genotype was associated with decreased risk of coronary heart disease and restenosis after percutaneous coronary intervention in Asian populations [8].

People with longer GTn repeats show an increased risk of CVD, enhanced atherosclerosis progression [20], and reduced left ventricle ejection fraction [21]. Also, the L allele is associated with a higher risk of acute kidney injury after cardiac surgery [22], and chronic kidney disease [18]. Two studies on ischemic stroke found that certain subgroups carrying the SS allele (< 25 repeats) had a reduced risk of stroke or transient ischemic attack [23, 24].

The AA genotype of the -413A/T polymorphism has been associated with lower risk of coronary artery disease [6, 8] but also with higher risk of hypertension in women [5]. Individuals with at least one A allele had better outcomes after an atherosclerotic stroke than those with the TT genotype [7].

Plasma HO-1 concentrations have been reported to be elevated in stable coronary artery disease and in acute coronary artery syndromes [25, 26]. Also, in chronic heart failure the HO-1 plasma concentrations increase along with the NYHA class [27]. Furthermore, individuals with stroke had higher serum concentrations of HO-1 compared to those with transient ischemic attacks [28].

Diabetes increases all-cause mortality significantly [29], but it is of note that 44% of the deaths in T1D and 52% in type 2 diabetes (T2D) are caused by CVD. Kidney disease accounts for 21% of deaths in T1D and 11% in T2D. Age-adjusted mortality rates are generally higher in individuals with T1D compared to those with T2D [30]. Notably, only a few studies have addressed the role of HO-1 or the *HMOX1* gene polymorphism in cardiovascular complications in diabetes, and these studies have focused on T2D or gestational diabetes but not T1D.

HO-1 expression was found to be reduced in peripheral blood mononuclear cells of individuals with diabetes [31]. Interestingly, high expression levels of HO-1 in early pregnancy were associated with reduced risk of gestational diabetes [32]. Individuals with elevated plasma HO-1 concentrations had a higher risk of developing T2D [33]. In addition, people with the LL genotype had a higher risk of T2D or impaired glucose regulation, and those with diabetes carrying the LL genotype showed reduced HO-1 expression [31]. Given the associations between the genetic variants and the risk of cardiovascular and kidney disease, the inverse relationship between HO-1 expression and diabetes, and the association between plasma HO-1 concentrations and risk of T2D, the question arises, what is the role of the genetic variants and the HO-1 serum concentrations for the risk of cardiovascular and kidney disease in individuals with T1D.

Therefore, the aim of this study was to investigate the association of the *HMOX1* gene promoter area GTn length repeat, two different single nucleotide polymorphisms (− 413A/T (rs2071746) and + 99G/C (rs2071747), and serum HO-1 concentrations with vascular complications and all-cause mortality in individuals with T1D.

## The study participants and methods

### The study population

The individuals in this study are participants of the nationwide and multicenter FinnDiane Study that was launched in 1997. The FinnDiane Study is searching for genetic, clinical, environmental, and metabolic risk factors for diabetic complications. The study has been previously described in detail [34]. To date, over 5000 participants have been recruited.

At the study centers, data on participants with T1D (defined as age of onset before 40 years and insulin treatment initiated within 1 year of diagnosis) were collected during a routine visit to the attending physician by questionnaires, medical records, and a thorough clinical investigation. Measurements of body weight, height, waist, and hip circumferences, as well as blood pressure were performed by a trained nurse. Blood samples were drawn and analyzed for lipid profile, hs-CRP, HbA1c, and creatinine as well as for genetic analyses. Urine collections were performed either as an overnight or 24-h urine collection. Data on lifestyle (smoking, alcohol use) were also collected.

### Definition of primary endpoints

The primary endpoints were ischemic cardiac event, stroke, peripheral artery disease, severe diabetic retinopathy, diabetic kidney disease, and death. Data on endpoints were first identified from the standardized questionnaires at the study visits. Furthermore, the endpoints were identified from the Care Register for Health Care and the Causes of Death Register [35]. An ischemic cardiac event was defined as myocardial infarction (ICD-8/9 410–412, ICD-10 I21-I23) or coronary procedure (bypass grafting surgery or angioplasty based on the Nordic Classifications of Surgical Procedures). Stroke was either ischemic or hemorrhagic (ICD-8/9 430–434, ICD-10 I60-I64). Severe diabetic retinopathy was defined as laser treatment due to severe nonproliferative retinopathy, proliferative retinopathy, or diabetic maculopathy. Peripheral artery disease was defined as revascularization or amputation due to peripheral artery disease. Kidney status was classified according to UAER based on two out of three timed urine collections: normoalbuminuria UAER < 20 µg/min or < 30 mg/24 h; moderate albuminuria (microalbuminuria) ≥ 20 and < 200 µg/min or ≥ 30 and > 300 mg/24 h; and severe albuminuria (macroalbuminuria) ≥ 200 µg/min or ≥ 300 mg/24 h. End-stage kidney disease (ESKD) was defined as ongoing dialysis or having received a kidney transplant. Kidney disease progression during follow-up was defined as any shift to a higher albuminuria class or ESKD after the measurement of HO-1.

### HMOX1 polymorphisms

We genotyped the *HMOX1* GTn repeat (*n* = 4556) and extracted from available genome-wide genotyping data two single nucleotide polymorphisms (SNPs) (-413A/T upstream variant rs2071746, and + 99G/C p.Asp7Asn missense variant rs2071747; *n* = 4897). Genome-wide genotyping was performed with Illumina HumanCoreExome chips at the University of Virginia, with quality control and genotype imputation with the 1000 Genomes phase 3 reference population as described earlier [36]. Individuals under the age of 18, with uncertain T1D or unknown kidney status were excluded from the analysis whereupon 3990 participants were included in the analysis of the *HMOX1* GTn length polymorphism, and 4278 in the SNP analyses.

The size of the GTn repeat polymorphism in the promoter area of *HMOX1* was studied by fragment analysis as described by Kaartokallio et al. in 2014 [37]. The cutoff point for the S and L alleles was set to S < 30 and L ≥ 30 according to the allele distribution and earlier studies. Individuals homozygous for the L allele were compared with the individuals carrying the S allele (SS and SL versus LL).

### HO-1 serum concentrations

The HO-1 concentrations were measured from serum samples collected at the study visit and stored at -80 °C. We analyzed samples from 889 participants using a human HO-1 enzyme-linked immunosorbent assay kit according to the manufacturer’s instructions (Human Heme Oxygenase-1 ELISA Kit (ab207621), Abcam, Cambridge, UK). After excluding participants under the age of 18, with uncertain T1D or unknown kidney status, 861 participants were included in the analysis.

### Statistical analysis

The statistical analysis was performed using SPSS statistical software version 27 (IBM Corp., Armonk, NY, USA). Normally distributed continuous variables are shown as means ± SD and otherwise as medians with interquartile range. Categorial variables are given as percentages. One way ANOVA was used to analyze between-group differences for normally distributed variables and the Mann Whitney or Kruskall Wallis test for the variables with skewed distribution. Chi-square test was used for categorial variables. Associations between HO-1 concentrations and cardiovascular traits in the full cohort and men and women separately were adjusted for multiple correction using the Benjamini Hochberg method, applied in R (4.5.1). The cumulative progression rates and mortality were assessed by Kaplan–Meier survival curves and compared with the log rank test. The association between different genotypes and vascular complications and all-cause mortality were analyzed using multivariable Cox regression. Follow-up started from birth and onset of diabetes with respect to the genotypes, and from the baseline study visit for the HO-1 concentrations (prospective analyses), and ended at the time of first appearance of any of the outcomes studied, death, or at the end of 2017. A *p-*value of < 0.05 was considered statistically significant. Genotype deviation from the Hardy Weinberg equilibrium (HWE), and linkage disequilibrium (LD) between the SNPs and the GTn L allele were estimated in all samples with genotype data available with plink v. 1.07.

## Results

### Distribution of HMOX1 alleles

**Fig. 1:**
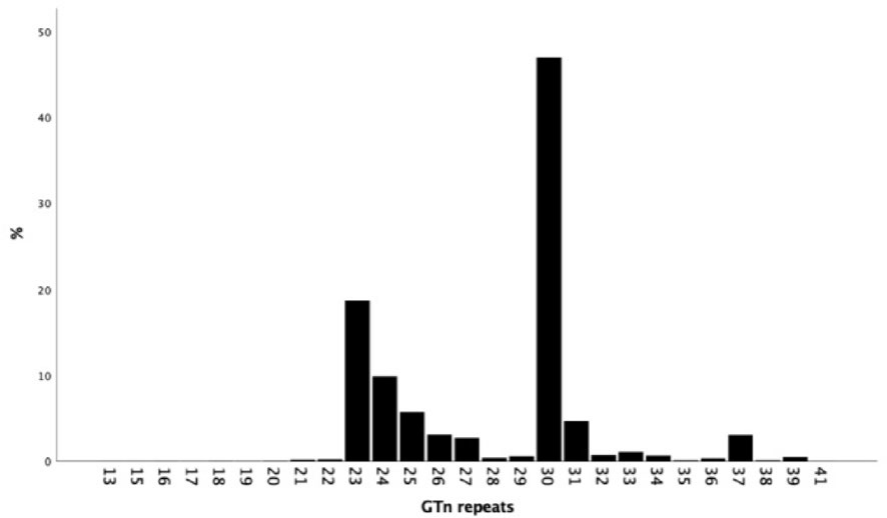
Distribution of *HMOX1* GTn repeats. Repeat lengths ≥ 30 were considered as L, i.e. long polymorphisms

The distribution of GTn repeats varied from 13 to 41, but the most frequent alleles were 23 (19.1%) and 30 (46,7%) (Fig. 1). From 3990 analyzed samples that were included in the analysis 17.7% had SS, 48.6% SL and 33.7% the LL genotype (cutoff L ≥ 30). The genotypes were in HWE (*p* = 1.00). In the analysis the S allele carriers (SS or SL genotype) were compared with the LL genotype (63.3% vs 33.7%). No differences in the laboratory findings or clinical characteristics were observed between SS/SL and LL genotypes (Table 1).

**Table 1:** Clinical and biochemical characteristics of study participants stratified by *HMOX1* GTn repeat genotype

	SS and SL	LL	*p* value
N = 3990	2645	1345	
Sex (men %)	51.7%	52.6%	0.598
Age (yrs)	38.3 ± 11.7	38.7 ± 11.8	0.351
Duration of diabetes (yrs)	22.4 ± 12.3	22.5 ± 12.2	0.896
BMI (kg/m^2^)	25.0 ± 3.6	25.0 ± 3.6	0.971
WHR men	0.917 ± 0.072	0.915 ± 0.072	0.717
WHR women	0.819 ± 0.064	0.821 ± 0.068	0.586
SBP (mmHg)	134.4 ± 18.5	135.2 ± 20.1	0.242
DBP (mmHg)	79.3 ± 9.8	79.7 ± 10.5	0.355
Hypertension (%)	39.3%	40.3%	0.531
HbA_1c_ (%)	8.5 ± 1.5	8.4 ± 1.5	0.533
HbA_1c_ (mmol/mol)	69.0 ± 16.3	68.6 ± 15.9	0.532
eGFR (ml/min/1.73 m2)	93.04 ± 30.72	91.32 ± 31.41	0.099
Insulin dose IU/kg	0.69 ± 0.26	0.70 ± 0.27	0.428
Total cholesterol (mmol/l)	4.92 ± 0.97	4.94 ± 1.01	0.519
HDL-cholesterol (mmol/l)	1.34 ± 0.39	1.33 ± 0.40	0.216
LDL-cholesterol (mmol/l)	3.05 ± 0.85	3.05 ± 0.88	0.853
APOA1 (mg/dl)	138.8 ± 23.3	137.9 ± 22.9	0.233
APOB (mg/dl)	87.0 ± 22.9	86.6 ± 23.0	0.643
Triglycerides (mmol/l)	1.04 (0.77–1.46)	1.03 (0.77–1.51)	0.697
hs-CRP (mg/l, values over 10 excluded)	2.47 ± 2.09	2.45 ± 1.96	0.744
HO-1 all (ng/ml) *n* = 804	2.83 ± 1.12 (*n* = 527)	2.91 ± 1.14 (*n* = 277)	0.366
HO-1 men (ng/ml) *n* = 379	3.04 ± 1.11 (*n* = 248)	3.22 ± 1.27 (*n* = 131)	0.150
HO-1 women (ng/ml) *n* = 425	2.65 ± 1.10 (*n* = 279)	2.62 ± 0.93 (*n* = 146)	0.840
AHT medication (%)	39.6%	40.8 (%)	0.468
Beta-blockers (%)	14.7%	15.4%	0.539
ACE-inhibitors (%)	25.6%	24.3%	0.363
AT1R blockers (%)	6.1%	5.9%	0.771
Calcium channel blockers (%)	12.2%	14.1%	0.108
Lipid lowering medication (%)	12.7%	13.4%	0.550
Diuretics (%)	13.0%	14.5%	0.181
NSAID use (%)	14.4%	15.8%	0.226
Ever-smokers (%)	47.1%	46.8%	0.844

Data are means ± SD, median (interquartile range) or percentages. *BMI* body mass index; *WHR* waist-hip ratio; *SBP* systolic blood pressure; *DBP* diastolic blood pressure; *HDL* high-density lipoprotein; HbA_1c_ glycated hemoglobin; *eGFR* estimated glomerular filtration rate; *LDL* low-density lipoprotein; *APOA1* apolipoprotein A-I; *APOB* apolipoprotein B; hs-CRP high-sensitivity C-reactive protein; HO-1 heme oxygenase 1; *AHT* antihypertensive; *ACE* angiotensin-converting enzyme; *AT1R* angiotensin II type 1 receptor; *NSAID* non-steroidal anti-Inflammatory drug. SS and SL vs. LL, L ≥ 30 GTn repeats

The − 413A/T and + 99G/C SNPs were determined for 4278 individuals included in the analysis. For − 413A/T, 31.8% were AA homozygous, 48.6% were heterozygous and 17.3% carried the TT genotype (HWE *p* = 0.52). For + 99G/C, 88.4% carried the GG genotype, 9.0% were heterozygous and only 0.3% were CC homozygous (HWE *p* = 0.44). The A allele of the -413A/T SNP was in LD with the L allele (most frequently 30 repeats) of the GTn polymorphism (*r*^*2*^ = 0.67, *D'* = 0.84). The estimated haplotype frequencies were 53.5% for A/L, 4.9% for T/L, 3.9% for A/S, and 37.7% for T/S. The + 99G/C variant was in low LD with the GTn polymorphism (*r*^*2*^ = 0.03, *D*′ = 0.90), as well as the − 413A/T and + 99G/C SNPs with each other (*r*^*2*^ = 0.07, *D*′ = 1.00).

### HMOX1 GTn microsatellite polymorphisms, vascular complications and all-cause mortality

**Fig. 2:**
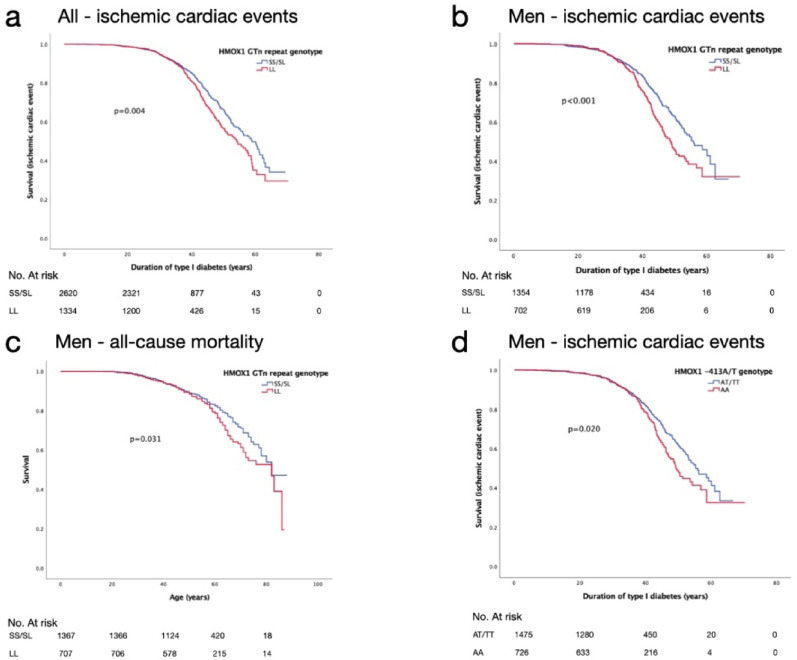
Kaplan–Meier curves for ischemic cardiac events and mortality according to *HMOX1* GTn repeat and -413A/T genotype. Curves for **a** ischemic cardiac events in all individuals, **b** ischemic cardiac events in men, **c** all-cause mortality in men, **d** Kaplan–Meier analysis for ischemic cardiac events stratified by *HMOX1* -413A/T SNP genotype in men

The LL genotype was associated with ischemic cardiac events during follow-up (LL 19.5% vs SS/SL 16%, *p* = 0.006, Table 2), also seen in the Kaplan–Meier survival curve with shorter event-free time from the onset of diabetes during a mean follow-up of 36.4 ± 0.2 years (*p* = 0.004, Fig. 2a; from birth p = 0.026). The association between the LL genotype and the ischemic cardiac events was even stronger among men (LL 22.9% vs SS/SL 17.0%, *p* = 0.001, Table 2), who had a shorter event-free time from the onset of diabetes during a mean follow up of 36.0 ± 0.3 years (*p* < 0.001, Fig. 2b; from birth *p* = 0.004). When analyzing women alone, there was no statistically significant difference in ischemic cardiac events (LL 15.7% vs SS/SL 14.9%, NS, Table 2). Men with the LL genotype had also higher all-cause mortality when stratified by the GTn genotype during a mean follow up of 54.9 ± 0.3 years (*p* = 0.031) (Fig. 2c). In women, there was no difference in the mortality rates. Separately plotted survival curves of SS, SL, and LL carriers are presented in Additional file 1. Pooling SS and SL carriers in the main results is supported by similar risk curves.

**Table 2:** Cardiovascular complications and severe diabetic retinopathy according to *HMOX1* GTn repeat genotype

	SS and SL events (%) (*n* = events/total)	LL events (%) (*n* = events/total)	*p* value
Ischemic cardiac event	16.0% (421/2627)	19.5% (261/1340)	**0.006**
Men	17.0% (231/1356)	22.9% (161/704)	**0.001**
Women	14.9% (190/1271)	15.7% (100/636)	NS (*p* = 0.657)
Stroke	9.7% (254/2628)	10.6% (142/1340)	NS (*p* = 0.354)
Men	11.7% (159/1356)	12.1% (85/704)	NS (*p* = 0.817)
Women	7.5% (95/1272)	9.0% (57/636)	NS (*p* = 0.256)
Peripheral artery disease	4.4% (117/2630)	5.7% (77/1340)	NS (*p* = 0.073)
Men	5.2% (70/1358)	7.3% (51/703)	NS (*p* = 0.055)
Women	3.7% (47/1272)	4.1% (26/637)	NS (*p* = 0.678)
Severe diabetic retinopathy	34.4% (900/2613)	37.5% (498/1329)	NS (*p* = 0.060)
Men	38.3% (517/1350)	41.2% (286/695)	NS (*p* = 0.211)
Women	30.3% (383/1263)	33.4% (212/634)	NS (*p* = 0.168)

Data are percentages. SS and SL vs. LL, L ≥ 30 GTn repeats

Bold values indicate statistically significant *p*-values (*p* < 0.05)

In multivariable Cox regression analysis, the association of the LL genotype with incident ischemic cardiac events remained significant when adjusted for study baseline BMI, triglycerides, HbA1c, systolic BP, stage of diabetic kidney disease, and duration of diabetes (HR (95% CI) 1.407 (1.138–1.739), *p* = 0.002 in men, HR (95% CI) 1.184 (1.007–1.393), *p* = 0.041 in all, Table 3). In men, the LL genotype was also associated with all-cause mortality in the Cox regression model (HR (95% CI) 1.316 (1.056–1.639), *p* = 0.014, Table 3).

**Table 3:** Hazard ratio (HR) for ischemic cardiac complications and all-cause mortality according to *HMOX1* GTn repeat and -413A/T genotypes

			Model 1		Model 2	
Variant	Outcome	Subgroup	HR (95% CI)	*p* value	HR (95% CI)	*p* value
GTn LL vs SS/SL	Ischemic cardiac events	All	1.192 (1.021–1.319)	0.026	1.184 (1.007–1.393)	**0.041**
		Men	1.341 (1.096–1.640)	0.004	1.407 (1.138–1.739)	**0.002**
		Women	1.013 (0.795–1.291)	0.917	0.946 (0.730–1.225)	0.674
	All-cause mortality	All	1.104 (0.944–1.290)	0.214	1.175 (1.000–1.380)	0.051
		Men	1.260 (1.020–1.555)	0.032	1.316 (1.056–1.639)	**0.014**
		Women	0.940 (0.744–1.187)	0.601	1.027 (0.808–1.305)	0.827
-413A/T AA vs AT /TT	Ischemic cardiac events	All	1.138 (0.977–1.326)	0.097	1.173 (0.999–1.377)	0.051
		Men	1.237 (1.012–1.511)	0.038	1.311 (1.063–1.618)	**0.011**
		Women	1.018 (0.801–1.290)	0.886	1.017 (0.789–1.311)	0.895
	All-cause mortality	All	1.038 (0.889–1.211)	0.641	1.111 (0.948–1.303)	0.194
		Men	1.175 (0.956–1.444)	0.126	1.232 (0.996–1.525)	0.054
		Women	0.891 (0.704–1.127)	0.335	0.982 (0.771–1.252)	0.886

Bold values indicate statistically significant *p*-values (*p* < 0.05)

Model 1: Unadjusted

Model 2: Adjusted for main cardiometabolic risk factors: age, sex, BMI, triglycerides, systolic blood pressure, HbA1c, stage of diabetic kidney disease, and duration of diabetes

The association of the *HMOX1* GTn length polymorphism with other primary endpoints such as stroke, diabetic kidney disease, peripheral artery disease or severe diabetic retinopathy was neither significant in men nor in women, although there was a trend suggesting that LL carriers had more events (Tables 2 and 4).

**Table 4:** Distribution of *HMOX1* GTn repeat genotypes in different categories of diabetic kidney disease

	No albuminuria (%)	Moderate albuminuria (%)	Severe albuminuria (%)	End-stage kidney disease (%)
SS/SL genotypes (*n* = 2645)	64.2%	13.2%	14.6%	8.0%
Men (*n* = 1367)	58.7%	14.6%	16.8%	9.8%
Women (*n* = 1278)	70.0%	11.7%	12.2%	6.1%
LL genotype (*n* = 1345)	62.7%	12.7%	15.8%	8.8%
Men (*n* = 707)	57.7%	15.0%	18.2%	9.1%
Women (*n* = 638)	68.2%	10.2%	13.0%	8.6%
*p* value	NS	NS	NS	NS

Data are percentages. SS and SL vs. LL, L ≥ 30 GTn repeats

### −413A/T (rs2071746) and + 99G/C (rs2071747), vascular complications and all-cause mortality

Men carrying the AA genotype experienced ischemic cardiac events more frequently compared with those carrying the AT/TT genotypes (21.0% vs 17.4%, *n* = 730 and *n* = 1476, respectively, *p* = 0.044) also shown in the Kaplan–Meier curve (event-free time from birth, *p* = 0.037 and from onset of diabetes *p* = 0.020 during a mean follow-up of 35.7 ± 0.3 years, Fig. 2d). In the Cox regression analysis, the AA genotype remained significantly associated with incident cardiovascular events in men when adjusted for BMI, triglycerides, HbA1c, systolic BP, stage of diabetic kidney disease, and duration of diabetes (HR (95% CI) 1.311 (1.063–1.618), *p* = 0.011, Table 3).

In women as well as in all together, there were no significant differences in ischemic cardiac events. In addition, regarding the other studied vascular and kidney complications or all-cause mortality, no differences were found between the AA vs AT/TT genotypes. There were no differences either in the laboratory or clinical findings between the different genotypes.

Concerning the + 99G/C SNP, no differences were found in all-cause mortality, cardiovascular and kidney complications, clinical measurements, or laboratory variables between the different genotypes. The small number of CC homozygous individuals makes the analysis partly unpowered.

### HO-1 serum concentrations

The HO-1 serum concentrations differed markedly between men and women (3.12 ± 1.23 ng/ml vs 2.64 ± 1.04 ng/ml, *n* = 411 and 450, respectively, *p* < 0.001). Therefore, in the statistical analysis, men and women were analyzed separately. Comparison between men and women regarding clinical findings, measurements, and treatment is presented in Additional file 2. There were no significant differences in the serum HO-1 concentrations between the different GTn, -413A/T or + 99G/C genotypes (data not shown).

HO-1 concentrations were higher among participants with a history of ischemic cardiac events, stroke, peripheral artery disease, hypertension, diabetic kidney disease, and severe diabetic retinopathy, especially in women (Table 5). Participants, and once again especially women taking antihypertensive medication, low-dose aspirin, or lipid-lowering medications had higher HO-1 concentrations (Table 5).

**Table 5:** Association of serum HO-1 concentrations with presence of vascular complications and need for medications

	HO-1 (ng/ml) No event / no medication	HO-1 (ng/ml) Event / medication	*p* value	FDR
Ischemic cardiac event	2.85 ± 1.15 (*n* = 810)	3.31 ± 1.34 (*n* = 45)	**0.008**	**0.016**
Men	3.11 ± 1.23 (*n* = 383)	3.38 ± 1.29 (*n* = 22)	NS (p = 0.329)	0.377
Women	2.61 ± 1.01 (*n* = 427)	3.26 ± 1.42 (*n* = 23)	**0.003**	**0.007**
Stroke	2.86 ± 1.17 (*n* = 834)	3.19 ± 0.98 (*n* = 22)	NS (*p* = 0.193)	0.228
Men	3.13 ± 1.24 (*n* = 389)	3.12 ± 1.04 (*n* = 17)	NS (p = 0.968)	0.968
Women	2.63 ± 1.04 (*n* = 445)	3.45 ± 0.75 (*n* = 5)	NS (p = 0.082)	0.110
Peripheral artery disease	2.82 ± 1.11 (n = 805)	3.71 ± 1.62 (*n* = 48)	< **0.001**	** < 0.005**
Men	3.10 ± 1.23 (*n* = 380)	3.47 ± 1.33 (*n* = 26)	NS (p = 0.144)	0.176
Women	2.57 ± 0.92 (*n* = 425)	3.99 ± 1.91 (*n* = 22)	< **0.001**	** < 0.005**
Severe diabetic retinopathy	2.78 ± 1.07 (*n* = 598)	3.08 ± 1.33 (*n* = 256)	< **0.001**	** < 0.005**
Men	3.04 ± 1.12 (*n* = 268)	3.30 ± 1.42 (*n* = 137)	**0.048**	0.067
Women	2.57 ± 0.98 (*n* = 330)	2.83 ± 1.19 (*n* = 119)	**0.018**	**0.031**
Diabetic kidney disease	2.77 ± 1.08 (*n* = 697)	3.33 ± 1.38 (*n* = 156)	** < 0.001**	** < 0.005**
Men	3.05 ± 1.18 (*n* = 312)	3.37 ± 1.36 (*n* = 95)	**0.027**	**0.042**
Women	2.54 ± 0.93 (*n* = 385)	3.26 ± 1.42 (*n* = 61)	** < 0.001**	** < 0.005**
Hypertension	2.75 ± 1.06 (*n* = 510)	3.06 ± 1.28 (*n* = 346)	** < 0.001**	** < 0.005**
Men	3.00 ± 1.11 (*n* = 225)	3.30 ± 1.36 (*n* = 181)	**0.014**	**0.025**
Women	2.55 ± 0.97 (*n* = 285)	2.80 ± 1.14 (*n* = 165)	**0.011**	**0.021**
ACE inhibitors	2.86 ± 1.20 (*n* = 668)	2.91 ± 1.01 (*n* = 182)	NS (p = 0.578)	0.626
Men	3.13 ± 1.28 (*n* = 299)	3.09 ± 1.11 (*n* = 101)	NS (p = 0.775)	0.795
Women	2.63 ± 1.08 (*n* = 369)	2.68 ± 0.83 (*n* = 81)	NS (p = 0.688)	0.725
AT1R blockers	2.82 ± 1.10 (*n* = 747)	3.21 ± 1.50 (*n* = 102)	**0.002**	**0.006**
Men	3.08 ± 1.16 (*n* = 347)	3.45 ± 1.65 (*n* = 52)	**0.043**	0.062
Women	2.60 ± 1.00 (*n* = 400)	2.96 ± 1.30 (*n* = 50)	**0.022**	**0.036**
Beta blockers	2.79 ± 1.09 (*n* = 709)	3.26 ± 1.39 (*n* = 141)	< **0.001**	** < 0.005**
Men	3.04 ± 1.17 (*n* = 324)	3.47 ± 1.45 (*n* = 76)	**0.006**	**0.013**
Women	2.58 ± 0.98 (*n* = 385)	3.01 ± 1.29 (*n* = 65)	**0.002**	**0.006**
Calcium channel blockers	2.83 ± 1.14 (*n* = 746)	3.18 ± 1.25 (*n* = 102)	**0.004**	**0.009**
Men	3.08 ± 1.21 (*n* = 333)	3.34 ± 1.37 (*n* = 65)	NA (*p* = 0.123)	0.155
Women	2.62 ± 1.05 (*n* = 413)	2.90 ± 0.95 (*n* = 37)	NA (*p* = 0.118)	0.153
Any AHT medication	2.73 ± 1.05 (*n* = 516)	3.08 ± 1.29 (*n* = 335)	< **0.001**	** < 0.005**
Men	2.99 ± 1.11 (*n* = 227)	3.30 ± 1.37 (*n* = 174)	**0.012**	**0.022**
Women	2.53 ± 0.95 (*n* = 289)	2.84 ± 1.16 (*n* = 161)	**0.003**	**0.007**
NSAIDs (low-dose ASA)	2.78 ± 1.10 (*n* = 651)	3.15 ± 1.31 (*n* = 201)	< **0.001**	** < 0.005**
Men	3.04 ± 1.20 (*n* = 297)	3.35 ± 1.30 (*n* = 105)	**0.031**	**0.047**
Women	2.57 ± 0.95 (*n* = 354)	2.93 ± 1.30 (*n* = 96)	**0.002**	**0.006**
Lipid-lowering medication	2.77 ± 1.11 (*n* = 658)	3.19 ± 1.26 (*n* = 192)	< **0.001**	** < 0.005**
Men	3.10 ± 1.22 (*n* = 292)	3.19 ± 1.28 (*n* = 109)	NS (*p* = 0.510)	0.568
Women	2.52 ± 0.94 (*n* = 366)	3.18 ± 1.28 (*n* = 83)	< **0.001**	** < 0.005**

Bold values indicate statistically significant *p*-values (*p* < 0.05)

Data are means ± SD. *ACE* angiotensin-converting enzyme; *AT1R* angiotensin II type 1 receptor; *AHT* antihypertensive; *NSAID* non-steroidal anti-inflammatory drug; *ASA* acetylsalicylic acid. *FDR* false discovery rate, calculated with the Benjamin Hochberg method

Kidney complications stratified by quartiles of serum HO-1 concentrations are shown in Table 6. The HO-1 concentrations were higher in individuals with lower eGFR and higher stage of albuminuria. Other laboratory and clinical measurements associated with the HO-1 quartiles included e.g., waist-hip-ratio, total cholesterol, and apolipoprotein A1 concentrations in men (Table 7).

**Table 6:** Kidney complications stratified by quartiles of serum HO-1 concentrations

	HO-1 1th quartile	HO-1 2nd quartile	HO-1 3rd quartile	HO-1 4th quartile	*p* value
eGFR (ml/min/1.73 m2)	104.75 ± 23.61 (*n* = 121)	104.24 ± 25.52 (*n* = 131)	103.54 ± 23.10 (*n* = 132)	84.92 ± 36.64 (*n* = 125)	** < 0.001**
Men	105.27 ± 28.25 (*n* = 50)	104.64 ± 30.18 (*n* = 67)	109.21 ± 20.12 (*n* = 64)	87.17 ± 39.05 (*n* = 56)	** < 0.001**
Women	104.39 ± 19.29 (*n* = 71)	103.81 ± 19.75 (*n* = 64)	98.20 ± 24.55 (*n* = 68)	83.10 ± 34.74 (*n* = 69)	< **0.001**
Albuminuria					All < **0.001** Women < **0.001** Men **0.04**
No albuminuria (*n* = 626)	29.1%	24.9%	25.6%	20.4%	
Men (*n* = 279)	28.0%	24.7%	28.0%	19.4%	
Women (*n* = 347)	30.0%	25.1%	23.6%	21.3%	
Moderate albuminuria (*n* = 84)	13.1%	29.8%	27.4%	29.8%	
Men (*n* = 41)	17.1%	29.3%	19.5%	34.1%	
Women (*n* = 43)	9.3%	30.2%	34.9%	25.6%	
Severe albuminuria (*n* = 70)	18.6%	18.6%	21.4%	41.4%	
Men (*n* = 43)	18.6%	18.6%	23.3%	39.5%	
Women (*n* = 27)	18.5%	18.5%	18.5%	44.4%	
ESKD (*n* = 78)	12.8%	25.6%	21.8%	39.7%	
Men (*n* = 45)	22.2%	26.7%	15.6%	35.6%	
Women (*n* = 33)	0.0%	24.2%	30.3%	45.5%	
Diabetic kidney disease progression after study baseline (*n* = 814)	6.7%	9.0%	4.9%	18.1%	< **0.001**
Men (*n* = 385)	6.1%	8.2%	6.0%	23.3%	< **0.001**
Women (*n* = 429)	5.5%	6.7%	8.4%	13.9%	NS (*p* = 0.129)

Bold values indicate statistically significant *p*-values (*p* < 0.05)

Data are means ± SD or percentages. *eGFR* estimated glomerular filtration rate; *ESKD* end-stage kidney disease

**Table 7:** Cardiovascular risk markers and clinical risk factors by serum HO-1 concentration quartiles

		HO-1 1th quartile	HO-1 2nd quartile	HO-1 3rd quartile	HO-1 4th quartile	*p* value
HS-CRP (mg/l) (values ≥ 10 excluded)	Men	0.98 (0.48 -1.75) (*n* = 48)	1.02 (0.57–1.92) (*n* = 63)	1.12 (0.62–1.99) (*n* = 59)	1.17 (0.68–3.10) (*n* = 47)	NS (*p* = 0.218)
	Women	1.35 (0.73–2.29) (*n* = 67)	1.57 (0.58–3.42) (*n* = 57)	1.55 (0.99–3.18) (*n* = 63)	1.50 (0.62–3.48) (*n* = 59)	NS (*p* = 0.508)
HbA1C (mmol/mol)	Men	61.38 ± 14.89 (*n* = 50)	63.03 ± 16.17 (*n* = 67)	63.29 ± 14.75 (*n* = 63)	64.63 ± 16.53 (*n* = 56)	NS (*p* = 0.765)
	Women	64.02 ± 12.51 (*n* = 70)	69.85 ± 16.03 (*n* = 64)	70.06 ± 17.03 (*n* = 68)	67.68 ± 15.31 (*n* = 68)	NS (*p* = 0.075)
Total cholesterol (mmol/l)	Men	4.17 ± 0.72 (*n* = 50)	4.49 ± 0.93 (*n* = 67)	4.52 ± 0.82 (*n* = 64)	4.63 ± 1.02 (*n* = 56)	**0.032**
	Women	4.59 ± 0.71 (*n* = 71)	4.71 ± 0.85 (*n* = 64)	4.69 ± 0.66 (*n* = 68)	4.66 ± 0.82 (*n* = 69)	NS (*p* = 0.826)
LDL-cholesterol (mmol/l)	Men	2.40 ± 0.62 (*n* = 31)	2.75 ± 0.83 (*n* = 53)	2.57 ± 0.73 (*n* = 56)	2.79 ± 0.81 (*n* = 42)	NS (*p* = 0.101)
	Women	2.65 ± 0.57 (*n* = 53)	2.53 ± 0.68 (*n* = 58)	2.65 ± 0.56 (*n* = 60)	2.55 ± 0.69 (*n* = 59)	NS (*p* = 0.603)
HDL-cholesterol (mmol/l)	Men	1.30 ± 0.30 (*n* = 50)	1.41 ± 0.26 (*n* = 67)	1.36 ± 0.30 (*n* = 64)	1.42 ± 0.36 (*n* = 56)	NS (*p* = 0.138)
	Women	1.59 ± 0.38 (*n* = 71)	1.67 ± 0.41 (*n* = 64)	1.66 ± 0.45 (*n* = 68)	1.67 ± 0.41 (*n* = 69)	NS (*p* = 0.532)
HDL2-C (mmol/l)	Men	0.54 ± 0.33 (*n* = 50)	0.52 ± 0.25 (*n* = 66)	0.43 ± 0.23 (*n* = 62)	0.57 ± 0.27 (*n* = 55)	**0.045**
	Women	0.69 ± 0.31 (*n* = 69)	0.71 ± 0.30 (*n* = 63)	0.74 ± 0.31 (*n* = 68)	0.77 ± 0.33 (*n* = 67)	NS (*p* = 0.481)
HDL3-C (mmol/l)	Men	0.799 ± 0.184 (*n* = 50)	0.901 ± 0.203 (*n* = 66)	0.926 ± 0.168 (*n* = 62)	0.851 ± 0.250 (*n* = 55)	**0.006**
	Women	0.89 ± 0.21 (*n* = 69)	0.96 ± 0.23 (*n* = 63)	0.92 ± 0.21 (*n* = 68)	0.91 ± 0.23 (*n* = 67)	NS (*p* = 0.259)
Triglycerides (mmol/l)	Men	1.09 (0.82–1.51) (*n* = 50)	0.89 (0.68–1.27) (*n* = 67)	1.00 (0.80–1.27) (*n* = 64)	0.97 (0.79–1.62) (*n* = 56)	NS (*p* = 0.143)
	Women	0.77 (0.59–0.97) (*n* = 71)	0.79 (0.59–1.19) (*n* = 64)	0.83 (0.64–1.16) (*n* = 68)	0.87 (0.72–1.28) (*n* = 69)	NS (*p* = 0.137)
APOA1 (mg/dl)	Men	121.46 ± 18.81 (*n* = 50)	126.74 ± 16.97 (*n* = 66)	127.94 ± 16.94 (*n* = 62)	136.78 ± 23.35 (*n* = 55)	< **0.001**
	Women	141.01 ± 26.90 (*n* = 70)	147.47 ± 29.14 (*n* = 64)	151.57 ± 28.77 (*n* = 68)	148.01 ± 25.50 (*n* = 68)	NS (*p* = 0.155)
APOB (mg/dl)	Men	74.00 ± 20.19 (*n* = 50)	81.44 ± 26.12 (*n* = 66)	80.76 ± 22.91 (*n* = 62)	82.55 ± 27.95 (*n* = 55)	NS (*p* = 0.282)
	Women	75.20 ± 18.47 (*n* = 70)	77.64 ± 24.06 (*n* = 64)	75.94 ± 15.70 (*n* = 68)	77.68 ± 22.98 (*n* = 68)	NS (*p* = 0.862)
Insulin dose (IU/kg)	Men	0.688 ± 0.346 (*n* = 102)	0.694 ± 0.294 (*n* = 101)	0.685 ± 0.278 (*n* = 103)	0.643 ± 0.236 (*n* = 98)	NS (*p* = 0.595)
	Women	0.644 ± 0.235 (*n* = 112)	0.610 ± 0.234 (*n* = 113)	0.643 ± 0.213 (*n* = 112)	0.621 ± 0.264 (*n* = 112)	NS (*p* = 0.639)
BMI (kg/m2)	Men	25.50 ± 3.30 (*n* = 102)	25.06 ± 3.44 (*n* = 101)	25.55 ± 3.67 (*n* = 103)	25.71 ± 3.99 (*n* = 98)	NS (*p* = 0.618)
	Women	25.43 ± 3.82 (*n* = 112)	24.84 ± 3.92 (*n* = 113)	25.16 ± 4.41 (*n* = 112)	24.50 ± 3.76 (*n* = 112)	NS (*p* = 0.332)
WHR	Men	0.924 ± 0.070 (*n* = 99)	0.917 ± 0.070 (*n* = 97)	0.931 ± 0.062 (*n* = 102)	0.946 ± 0.079 (*n* = 95)	**0.045**
	Women	0.837 ± 0.065 (*n* = 112)	0.841 ± 0.063 (*n* = 113)	0.840 ± 0.059 (*n* = 111)	0.842 ± 0.060 (*n* = 105)	NS (*p* = 0.944)
SBP (mmHg)	Men	141.20 ± 19.54 (*n* = 100)	140.60 ± 20.37 (*n* = 100)	140.30 ± 18.94 (*n* = 103)	143.88 ± 18.85 (*n* = 97)	NS (*p* = 0.556)
	Women	129.54 ± 16.26 (*n* = 113)	131.94 ± 17.91 (*n* = 113)	135.31 ± 18.47 (*n* = 112)	137.07 ± 20.74 (*n* = 110)	**0.011**
DBP (mmHg)	Men	76.23 ± 8.67 (*n* = 100)	78.48 ± 10.14 (*n* = 100)	77.56 ± 8.53 (*n* = 103)	79.62 ± 9.79 (*n* = 97)	NS (*p* = 0.072)
	Women	75.54 ± 9.46 (*n* = 113)	76.93 ± 8.69 (*n* = 113)	77.57 ± 9.36 (*n* = 112)	75.92 ± 8.80 (*n* = 110)	NS (*p* = 0.318)
PP (mmhg)	Men	64.97 ± 18.14 (*n* = 100)	62.12 ± 16.08 (*n* = 100)	62.74 ± 17.41 (*n* = 103)	64.26 ± 16.75 (*n* = 97)	NS (*p* = 0.620)
	Women	54.00 ± 15.02 (*n* = 113)	55.01 ± 14.30 (*n* = 113)	57.74 ± 17.59 (*n* = 112)	61.15 ± 20.17 (*n* = 110)	**0.008**
BAI (%)	Men	23.06 ± 2.74 (*n* = 99)	23.10 ± 3.18 (*n* = 97)	23.50 ± 3.70 (*n* = 102)	23.88 ± 3.52 (*n* = 95)	NS (*p* = 0.267)
	Women	29.14 ± 4.92 (*n* = 112)	28.67 ± 4.66 (*n* = 113)	28.78 ± 4.61 (*n* = 111)	28.48 ± 4.74 (*n* = 105)	NS (*p* = 0.770)

Bold values indicate statistically significant *p*-values (*p* < 0.05)

Data are means ± SD or median (IQR). *WHR* waist-hip ratio; *SBP* systolic blood pressure; *DBP* diastolic blood pressure; *PP* pulse pressure; *BAI* body adiposity index

**Fig. 3:**
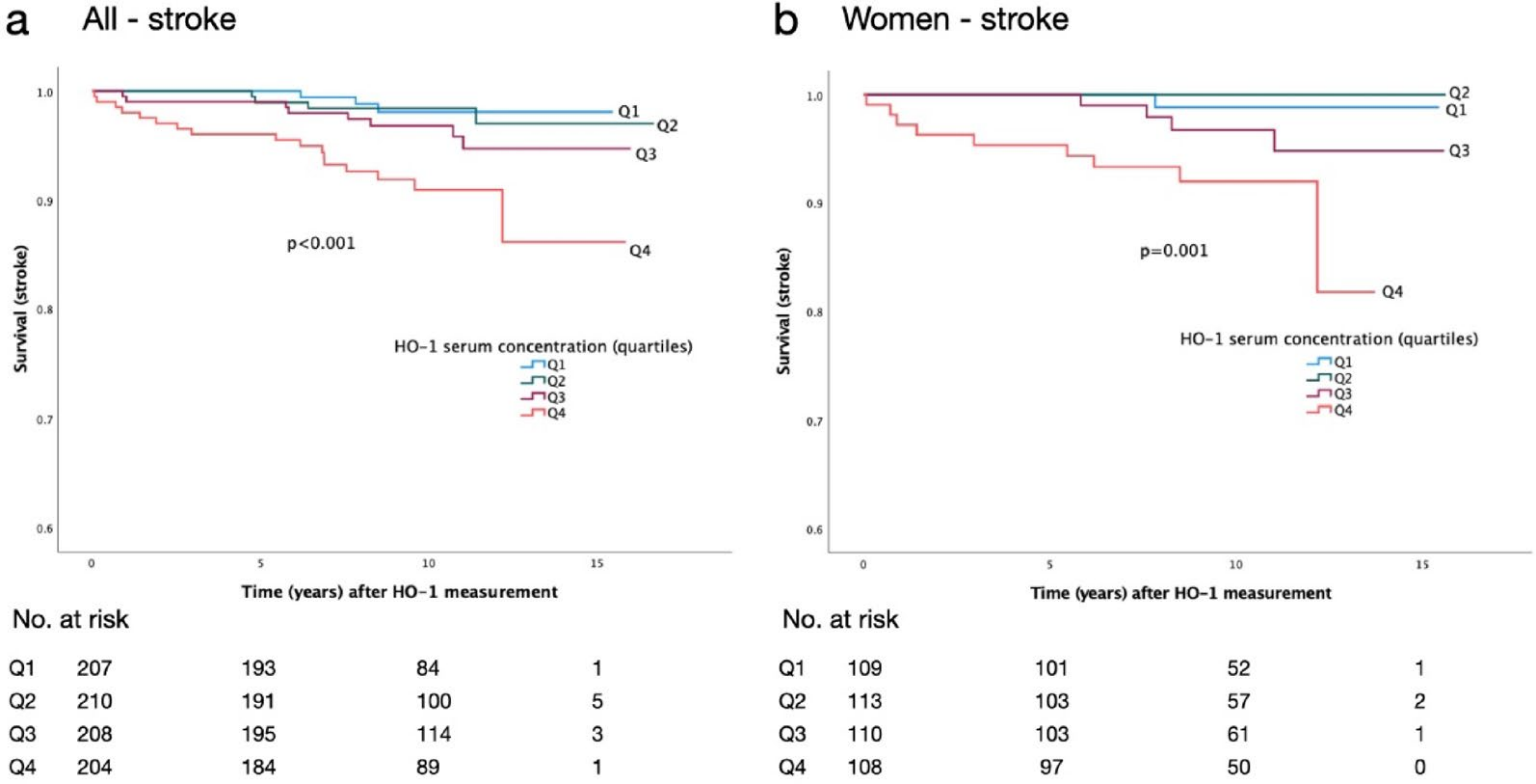
Kaplan–Meier curves for stroke according to quartiles of serum HO-1 concentrations. In **a** all individuals and **b** women

In the prospective analysis, a higher HO-1 serum concentration was associated with incident stroke in the entire study population (*p* < 0.001, mean follow-up time 9.7 ± 0.1 years) and in women separately (*p* = 0.001, mean follow-up time 9.7 ± 0.1 years) as seen in the Kaplan–Meier analysis (Fig. 3). Higher HO-1 concentrations were also associated with the progression of diabetic kidney disease (Table 6). There was no significant association between serum HO-1 concentrations and incident ischemic cardiac events or all-cause mortality.

## Discussion

In the present study, in individuals with T1D, the *HMOX1* gene promoter area GTn length polymorphism LL genotype was associated with ischemic cardiac events as compared with the SS/SL genotype. In men, the LL genotype was also associated with an increased risk of mortality. The HO-1 pathway acts cardioprotectively by several mechanisms, mediated by its by-products CO, biliverdin, and free iron [38]. The polymorphisms of the *HMOX1* gene, especially the GTn length polymorphism, influence its transcriptional activity. Therefore, several studies have evaluated whether people carrying varying *HMOX1* gene polymorphisms have different outcomes with respect to cardiovascular diseases [5, 6, 13, 18, 21, 24].

The key pathophysiological features of T1D include chronic hyperglycemia, oxidative stress, high levels of pro-inflammatory cytokines, increased glycemic variability leading to endothelial dysfunction, and metabolic disturbances that adversely affect lipid profiles and inflammation. T1D increases the demand for the cytoprotective effects of HO-1. In addition, the functional consequences of *HMOX1* polymorphisms may be more pronounced in patients with T1D.

In the previous studies, only limited data were available regarding the role of *HMOX1* gene polymorphism in people with diabetes. In a study involving 1474 individuals with newly diagnosed T2D or impaired glucose regulation, the LL genotype was associated with increased odds of impaired glucose regulation or diabetes compared with the SS or SL genotype [31]. To the best of our knowledge, the present study is the first to evaluate cardiovascular outcomes related to the *HMOX1* gene polymorphism in a large cohort of individuals with T1D.

Several studies have explored the association of GTn length polymorphisms with cardiovascular events in non-diabetic individuals. In agreement with our data in T1D, Zhang and coworkers found in their meta-analysis that the LL genotype was associated with coronary heart disease in non-diabetic individuals [8]. However, the association was found only in Asian populations but not in the Caucasian subgroup. Corresponding findings were also reported in another meta-analysis showing higher odds for cardiovascular disease in individuals having the LL genotype compared with the SL or SS genotype in Asian populations but not in the Caucasian population [12]. In a prospective, population-based study, individuals with the LL genotype had an increased risk of CVD and atherosclerosis progression [20]. In the Caucasians, higher all-cause mortality was reported in a primary prevention cohort study (n = 4937) in individuals carrying the LL allele compared with those carrying the SS allele [39].

In this study, we additionally analyzed two SNPs, the -413A/T upstream variant rs2071746 and the + 99G/C p.Asp7Asn missense variant rs2071747, and their association with cardiovascular events in individuals with T1D. We found that the AA genotype of the -413A/T SNP was associated with ischemic cardiac complications in men, while there were no differences in cardiovascular complications between the different + 99G/C genotypes. Notably, associations between these SNPs and cardiovascular complications have not been found in any cohorts with diabetes. Thus, in the study by Song and coworkers, no significant associations were found with the presence of T2D or impaired glucose regulation for the -413A/T polymorphisms [31]. Contrary to our results in individuals with diabetes, the AA genotype has been associated with a lower incidence of coronary artery disease in a non-diabetic Asian population [6] and in perimenopausal women [9]. On the other hand, a higher incidence of hypertension was found in women carrying the AA genotype [5]. Thus, the data on the -413A/T polymorphisms and CVD are not only scarce but also conflicting and appear to depend on the population studied. The functional significance of our present finding, that both the LL and the AA genotypes associate with ischemic cardiac events, warrants further studies as the A allele is in LD with the L allele in the studied population (*r*^*2*^ = 0.67, *D'* = 0.838 estimated in our study), and therefore may reflect the same association signal. LD between the A and L alleles was also previously reported in a Finnish cohort by Saukkonen et al. [11]. The GTn length polymorphism also overlaps two rs identifiers, rs3074372 and rs58433947, but due to the complexity of the GTn length polymorphism, the variant is not included, e.g., in the 1000 Genomes reference panel, commonly used for variant imputation or LD calculation, explaining also the challenges in investigating this locus.

The difference between men and women regarding the effect of *HMOX1* GTn and -413A/T polymorphisms on cardiovascular complications and all-cause mortality is interesting. When men and women were analyzed separately, the LL and AA genotypes were associated with ischemic cardiac events only in men, while no significant differences were found in women. An explanation for this sex difference is not clear from the present data. Differences in the inducibility and effect of the HO-1 system in cardiovascular and metabolic diseases have been discovered in experimental studies between male and female rodents. The HO-1 pathway has been found to be more inducible in females, which may even influence outcomes related to different genotypes [40–43]. However, our results suggest that *HMOX1* GTn and -413A/T polymorphisms may influence cardiovascular events and all-cause mortality in a sex-dependent manner.

There were no significant differences in the serum HO-1 concentrations between the different GTn, -413A/T or + 99G/C genotypes. The HO-1 concentrations were higher among men compared with women, and the higher concentrations were also associated with cardiovascular and kidney disease. This could be explained by CVD being more prevalent in men. Interestingly, in women, higher HO-1 concentrations were more strongly associated with cardiovascular events. Thus, it is possible that estrogens play a role in the inducibility of HO-1 in women and could be one explanation for the higher HO-1 concentrations in women with CVD [44]. HO-1 concentrations were also higher in the individuals using antihypertensive medication, acetylsalicylic acid (ASA), or lipid-lowering medication. The higher HO-1 concentration in these individuals could be partly explained by the drug effects, as ASA and statins have been shown to induce HO-1 [45–47]. The + 99 G/C variant C allele is shown to be highly associated with higher *HMOX1* gene expression [48]. Such an association was not observed in the present study, probably due to the small number of CC homozygous individuals; similarly, the lack of association between the GTn and -413A/T variants, and the HO-1 concentration may be explained by limited statistical power. It is likely that the serum HO-1 concentrations may merely reflect the presence of cardiovascular or kidney disease, whereas the *HMOX1* polymorphisms may influence cardiovascular outcomes independently of the serum HO-1 concentrations.

There are some further limitations in this study, especially regarding the interpretation of the serum HO-1 concentrations. The HO-1 concentrations were analyzed from samples taken during the participants’ regular visits, but the number of samples stored at -80 °C was limited, since the majority of the samples had been stored at -20 °C. In this study setting it is not unexpected that there were no differences in the HO-1 serum concentrations between the different genotypes, probably due to the high inducibility of HO-1 by several mechanisms. The HO-1 pathway is induced in individuals with cardiovascular and kidney disease, possibly even years before the stroke or cardiac ischemic events. Therefore, the HO-1 results in this study are more descriptive in character, and further studies are required to explore the mechanisms and pathogenetic significance of the HO-1 pathway in CVD in individuals with T1D.

The strength of this study is that it comprises a large, representative, and prospective cohort of 3990 individuals with T1D, of a homogenous Finnish origin. Moreover, the evaluation of cardiovascular and kidney disease and mortality data were carefully performed utilizing validated registry data [35]. Thus, this is the first large-scale study in individuals with T1D assessing *HMOX1* GTn repeats, -413A/T and + 99G/C SNPs, and their association with cardiovascular and kidney events. The size of the population also allowed us to analyze women and men separately.

Cardiovascular and kidney complications are the major cause of mortality and morbidity in individuals with T1D. However, there has been a downward trend in mortality and the prevalence CVD, especially among individuals with diabetes due to better glycemic control and management of cardiovascular risk factors as well as the introduction of new antidiabetic drugs with positive impact on cardiovascular and kidney prognosis [49–52]. Nevertheless, there is still a significant residual risk of CVD events left, and thus additional organ-protective medications are needed to prevent and treat the CVD in both individuals with or without diabetes [53]. Research on HO-1 and its end-products is active, aiming at utilizing their cytoprotective, anti-inflammatory, antioxidative, vasodilative, anticoagulative, and antiapoptotic properties [3, 54]. It has been suggested that either induction of the HO-1 pathway or direct application of its end-products could serve as additional future treatment options.

In conclusion, the LL genotype of the *HMOX1* gene was associated with ischemic cardiac complications in individuals with T1D in the present study. In men, the LL genotype was also associated with higher risk of mortality, and from the SNPs studied, the AA genotype of the -413A/T SNP was connected to ischemic cardiac events. Thus, the *HMOX1* LL GTn and/or the -413A/T SNP genotypes could be utilized as possible risk markers for coronary heart disease, especially in men. The serum HO-1 concentrations were higher in men compared to women, and higher concentrations were found to be associated with the presence of cardiovascular or kidney disease. This, in turn, suggests that serum HO-1 concentrations could serve as a possible tool to identify cardiovascular and kidney complications in individuals with T1D. Altogether, more research is warranted to understand the role and the potential clinical applications of the HO-1 pathway in CVD.

## Supplementary Information

Below is the link to the electronic supplementary material.

Supplementary Material 1

Supplementary Material 2

## Data Availability

Individual-level data for the study participants are not publicly available because of the restrictions due to the study consent provided by the participant at the time of data collection.

## Abbreviations

ACE: Angiotensin-converting enzyme
AHT: Antihypertensive
APOA1: Apolipoprotein A-I
APOB: Apolipoprotein B
ASA: Acetylsalicylic acid
AT1R: Angiotensin II type 1 receptor
BMI: Body mass index
CVD: Cardiovascular disease
DBP: Diastolic blood pressure
HbA_1c_: Glycated hemoglobin
eGFR: Estimated glomerular filtration rate
ESKD: End-stage kidney disease
FDR: False discovery rate
GTn: Guanine-thymine dinucleotide repeat
HDL: High-density lipoprotein
HO: Heme oxygenase
HO-1: Heme oxygenase 1
HO-2: Heme oxygenase 2
hs-CRP: High-sensitivity C-reactive protein
L: Long
LD: Linkage disequilibrium
LDL: Low-density lipoprotein
M: Medium
NSAID: Non-steroidal anti-Inflammatory drug
NYHA: New York Heart Association
S: Short
SBP: Systolic blood pressure
SNP: Single nucleotide polymorphism
T1D: Type 1 diabetes
T2D: Type 2 diabetes
UAER: Urinary albumin excretion rate
