# Supplementary material for: Heme oxygenase-1 polymorphisms associate with ischemic cardiac complications and all-cause mortality in type 1 diabetes
Source: Cardiovasc Diabetol. 2025 Aug 18;24:339. doi: 10.1186/s12933-025-02895-2 (PMC12363068; doi:10.1186/s12933-025-02895-2)
Supplement: Supplementary file 2 — Supplementary Material 2 [file 12933_2025_2895_MOESM2_ESM.docx]

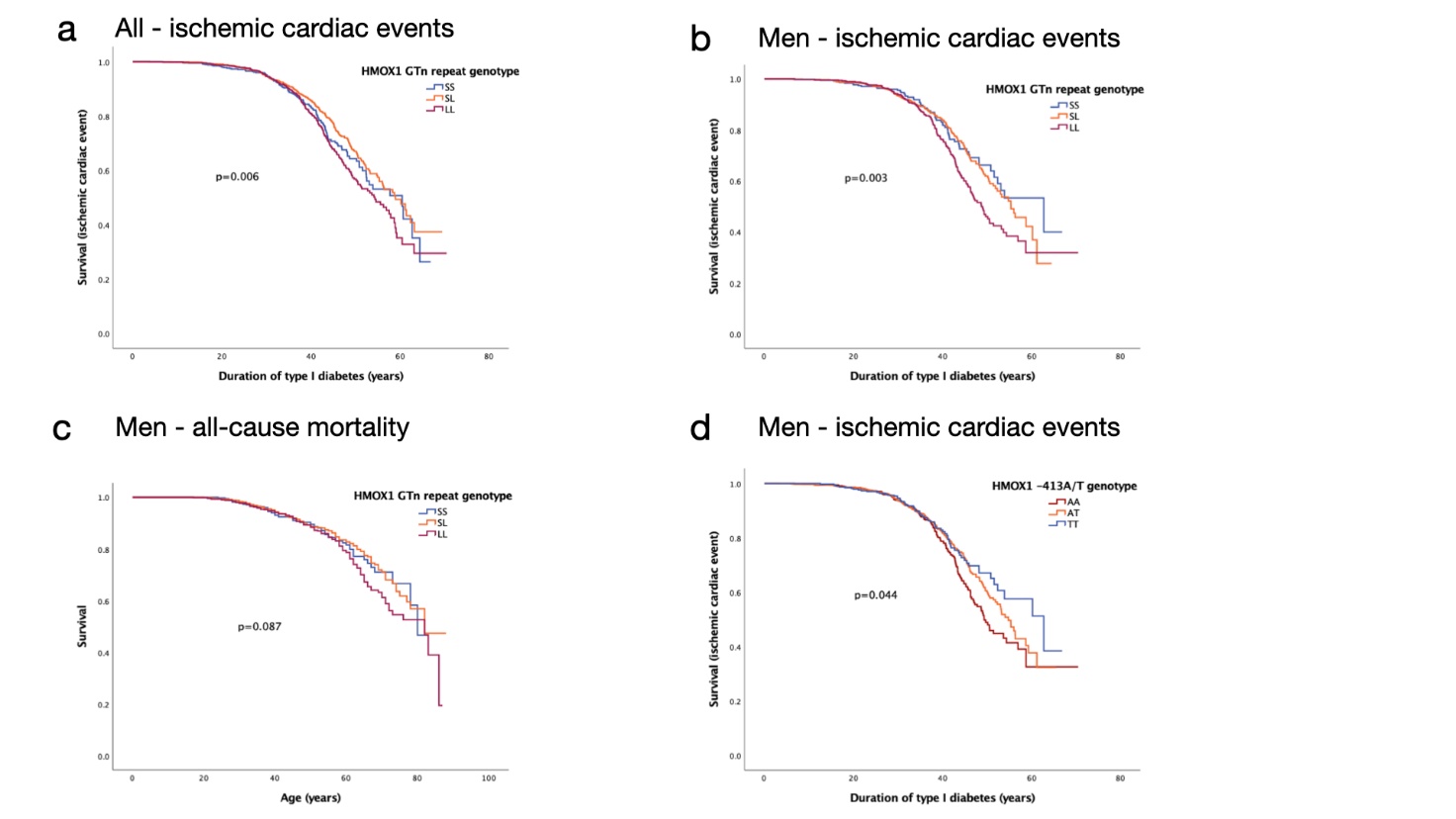


**Additional file 1.** **Kaplan–Meier curves for ischemic cardiac events and mortality according to *HMOX1* GTn repeat (SS, SL and LL separated) and -413A/T genotype.** Curves for **a)** ischemic cardiac events in all individuals, **b)** ischemic cardiac events in men, **c)** all-cause mortality in men, **d)** Kaplan–Meier analysis for ischemic cardiac events stratified by *HMOX1* -413A/T SNP genotype in men, AA, AT and TT separated.

**Additional file 2.** Clinical and biochemical characteristics of study participants included in serum HO-1 level analysis, men vs women.

|  | **Men** | **Women** | *p* value |
| --- | --- | --- | --- |
| **HS-CRP** (mg/l)  (values > 10 excluded) | 1.82±1.75  *n*=366 | 2.44±2.13  *n*=400 | <0.001 |
| **HbA1C** (mmol/mol) | 63.47±14.66  *n*=406 | 67.68±14.98  *n*=444 | <0.001 |
| **Total cholesterol** (mmol/l) | 4.68±0.95  *n*=408 | 4.77±0.85  *n*=449 | 0.130 |
| **LDL-cholesterol** (mmol/l) | 2.89±0.86  *n*=340 | 2.77±0.77  *n*=402 | 0.059 |
| **HDL-cholesterol** (mmol/l) | 1.30±0.34  *n*=408 | 1.57±0.40  *n*=449 | <0.001 |
| **HDL2-C** (mmol/l) | 0.49±0.26  *n*=391 | 0.69±0.29  *n*=439 | <0.001 |
| **HDL3-C** (mmol/l) | 0.82±0.21  *n*=391 | 0.89±0.22  *n*=439 | <0.001 |
| **Triglycerides** (mmol/l) | 1.00 (0.79-1.40)  *n*=408 | 0.86 (0.67-1.19)  *n*=449 | <0.001 |
| **APOA1** (mg/dl) | 129.39±19.94  *n*=395 | 146.90±25.22  *n*=442 | <0.001 |
| **APOB** (mg/dl) | 83.44±23.93  *n*=395 | 78.98±20.85  *n*=442 | 0.004 |
| **Insulin dose** (IU/kg) | 0.68±0.29  *n*=404 | 0.63±0.24  *n*=449 | 0.008 |
| **eGFR** | 97.41±31.33  *n*=410 | 93.97±27.37  *n*=449 | 0.086 |
| **BMI** (kg/m2) | 25.45±3.60  *n*=404 | 24.98±3.99  *n*=449 | 0.073 |
| **WHR** | 0.071±0.0036  *n*=393 | 0.062±0.0029  *n*=441 | <0.001 |
| **SBP** (mmHg) | 141.47±19.42  *n*=400 | 133.44±18.57  *n*=448 | <0.001 |
| **DBP** (mmHg) | 77.96±9.34  *n*=400 | 76.49±9.09  *n*=448 | 0.021 |
| **PP** (mmhg) | 63.51±17.09  *n*=400 | 56.95±17.07  *n*=448 | <0.001 |
| **BAI** (%) | 23.38±3.31  *n*=393 | 28.78±4.72  *n*=441 | <0.001 |
| **Ischemic cardiac event** (%) | 6.9  *n*=406 | 6.0  *n*=449 | 0.599 |
| **Stroke** (%) | 4.2  *n*=406 | 1.1  *n*=450 | 0.005 |
| **Peripheral artery disease** (%) | 6.4  *n*=406 | 4.9  *n*=447 | 0.348 |
| **Severe diabetic retinopathy** (%) | 33.8  *n*=405 | 26.5  *n*=449 | 0.020 |
| **Diabetic kidney disease** (%) | 23.3  *n*=407 | 13.7  *n*=446 | <0.001 |
| **Hypertension** (%) | 44.6  *n*=406 | 36.7  *n*=450 | 0.018 |
| **ACE inhibitors** (%) | 25.3  *n*=400 | 18.0  *n*=450 | 0.010 |
| **AT1R blockers** (%) | 13.0  *n*=399 | 11.1  *n*=450 | 0.390 |
| **Beta blockers** (%) | 19.0  *n*=400 | 14.4  *n*=450 | 0.075 |
| **Calcium channel blockers** (%) | 16.3  *n*=398 | 8.2  *n*=450 | <0.001 |
| **Any AHT medication** (%) | 43.4  *n*=401 | 35.8  *n*=450 | 0.023 |
| **NSAIDs (low-dose ASA)** (%) | 26.1  *n*=402 | 21.3  *n*=450 | 0.100 |
| **Lipid-lowering medication** (%) | 27.2  *n*=401 | 18.5  *n*=449 | 0.002 |

Data are means± SD, percentages or median (IQR). *WHR* waist-hip ratio; *SBP* systolic blood pressure; *DBP* diastolic blood pressure; *PP* pulse pressure; *BAI* body adiposity index. *ACE* angiotensin-converting enzyme; *AT1R* angiotensin II type 1 receptor; *AHT* antihypertensive; *NSAID* non-steroidal anti-inflammatory drug; *ASA* acetylsalicylic acid
